# The relationship between physical performance and alcohol consumption levels in Russian adults

**DOI:** 10.1038/s41598-024-51962-3

**Published:** 2024-01-16

**Authors:** Nikita A. Mitkin, German E. Kirilkin, Tatiana N. Unguryanu, Sofia Malyutina, Sarah Cook, Alexander V. Kudryavtsev

**Affiliations:** 1 Department of Community Medicine, UiT The Arctic University of Norway, N-9037 Tromsø, Norway; 2 International Research Competence Centre, Northern State Medical University, Troitsky Ave., 51, Arkhangelsk, Russia 163069; 3 Department of Hygiene and Medical Ecology, Northern State Medical University, Troitsky Ave., 51, Arkhangelsk, Russia 163069; 4 Research Institute of Internal and Preventive Medicine, Branch of Institute of Cytology and Genetics, Siberian Branch of the Russian Academy of Sciences, B.Bogatkova Str., 175/1, Novosibirsk, Russia 630089; 5 Department of Therapy, Hematology and Transfusiology, Novosibirsk State Medical University, Krasny Prospect, 52, Novosibirsk, Russia 630091; 6 School of Public Health, Imperial College London, White City Campus, 80-92 Wood Lane, London, W12 0BZ UK

**Keywords:** Diseases, Risk factors

## Abstract

Investigating the relationship between alcohol consumption and physical performance, we used data from the 2015–2018 Know Your Heart study on 4215 adults aged 35–69 from Arkhangelsk and Novosibirsk, Russia. We classified participants’ drinking status into non-drinking, non-problem drinking, hazardous drinking, and harmful drinking based on their self-reported drinking behaviors. To evaluate physical performance, we developed a Composite Physical Performance Scale (CPPS), which combined the results of three functional tests: grip strength (GS), closed-eyes balance, and chair rises (CR). We applied multivariable linear regression to assess the relationship between alcohol consumption and CPPS score, and ordinal logistic regression to explore the associations between alcohol consumption and the three functional tests separately. The results showed that harmful drinking was associated with lower CPPS scores compared to non-problem drinking. Among harmful drinking men, the decrease in CPPS scores was explained by all three tests equally and exceptionally by GS among women. Non-drinking was also associated with decreased CPPS, linked to lower GS and CR scores in men, and only lower GS scores in women. The study revealed a reduced physical performance in the non-drinking and harmful drinking groups compared to non-problem drinking.

## Introduction

Alcohol consumption is a global concern because of its high prevalence and negative impact on human health^1,2^. It increases risks of cardiovascular and liver diseases, cancer, infections, and injuries^1,3–5^. However, the effect of alcohol consumption on physical function, an essential component of an active and independent life, has not been thoroughly investigated^6–10^.

Physical function involves the ability to maintain postures and perform movements^11,12^ and is influenced by various factors such as age, sex, genetics, lifestyle, including nutrition, alcohol consumption, and others^13^. As people get older, diminished physical function may lead to low quality of life and increased risks of injury, morbidity, and mortality^14–16^. Given the global trend of aging, maintaining physical performance is a pressing public health matter^17,18^.

Research findings regarding the relationship between alcohol and physical performance are controversial^19–23^. Some studies suggest a positive or no association^7,24–27^, while others report negative effects, primarily in people who drink heavily^22,28^. The mechanisms of alcohol effects on health, the critical threshold for negative effects, and chronic long-term consequences of drinking on physical performance are still not fully described^9,10,29,30^. Besides, there is scarce knowledge of the variability in alcohol impact on physical performance in diverse socioeconomic and drinking habits contexts^28,31,32^.

Russia has one of the highest levels of alcohol consumption in the world with predominant spirits and binge-drinking pattern^33,34^. The COVID-19 pandemic and the economic crisis during 2020–2022 have increased alcohol consumption^35,36^. Given population aging in Russia the uncontrolled effects of the widespread alcohol consumption on physical function may potentially increase the burden of disease and disability among the elderly^37^. Therefore, exploring modifiable risk factors, such as alcohol consumption, is a public health priority to promote healthy aging^38^.

In light of these considerations, our study aims to investigate the relationship between alcohol consumption and physical performance in the Russian adult population.

## Materials and methods

### Study design and subjects

We used data from the Know Your Heart (KYH) cross-sectional study, part of the International Project on Cardiovascular Disease in Russia, conducted in Arkhangelsk and Novosibirsk from 2015 to 2018.

The KYH study population comprised 4,542 men and women aged 35–69 years, randomly selected from anonymized address databases of residents in four city districts of both Arkhangelsk and Novosibirsk, stratified by age, sex, and district. Trained interviewers attempted to reach randomly selected households up to five times to identify eligible participants. One household member who matched the specified age group and sex was invited to participate in the study. The study included a baseline interview at participants’ homes, and a health check at a polyclinic. The response proportion for the baseline interview was 68% in Arkhangelsk and 41% in Novosibirsk, out of total invitees of the required age and sex living at the selected addresses. Of these people 96% in Arkhangelsk and 83% in Novosibirsk underwent the health check.

The current study included 4215 KYH participants with complete data on alcohol consumption and physical performance assessments. Further details on the KYH study’s design and rationale can be found in a publication by Cook et al.^39^.

### Data collection

Data collection began with a baseline interview at participants’ homes using a computer-assisted personal interview tablet. The survey collected information on demographic, socioeconomic, behavioral, and health-related characteristics.

Alcohol consumption in the preceding 12 months was assessed by recording the frequency and volume of alcoholic beverages consumed; by using the Cutting down, Annoyance by criticism, Guilty feeling, and Eye-openers (CAGE) test^40^; and by questioning signs of harmful drinking pattern such as episodes of “zapoi” (drinking alcohol for two or more days, during which the person disconnects from normal social life), hangover, and excessive drunkenness. Participants also completed the EPIC questionnaire^41–43^ to assess physical activity and the Dietary Quality Score questionnaire to evaluate dietary quality^44^.

The health check involved a medical interview, physical examination, anthropometry, and biological sample collection.

The medical interview covered medical history, health-related behaviors, and included the Alcohol Use Disorders Identification Test (AUDIT) questionnaire^45–48^.

The physical examination included assessments of physical performance using grip strength, standing balance (with eyes open and closed), and chair rises tests adhering to standard protocols. Grip strength was measured by use of Jamar + Digital Dynamometer. Balance was assessed by recording the time a participant could stand on one leg, first with eyes open and then closed. Chair rises test required a participant to rise from a seated position ten times with time taken for the task and/or the number of chair rises completed recorded. The details of each test are provided in Supplementary Table S1. Participants had the right to refuse to participate in any of the tests for any reason. If a participant was unable to stand or walk unaided, balance and chair rise tests were not conducted.

The anthropometric data collection included measurements of height and weight using a Seca® 217 Stadiometer and a TANITA BC 418 Body Composition Analyzer, respectively. Each measurement was conducted twice, and the average was used for analysis.

Blood samples were collected at least 4 h after the participant’s last meal. Carbohydrate-deficient transferrin (CDT) levels were measured using capillary electrophoresis on the CAPILLARYS-2 system (Sebia S.A., France) for a subgroup of participants (N = 1670). Serum gamma-glutamyltransferase (GGT) levels were measured for all study participants using a kinetic color test on the AU 680 Chemistry System (Beckman Coulter).

### Outcome variable

To assess physical performance in this study, we developed a Composite Physical Performance Scale (CPPS), integrating score values from grip strength (GS), closed-eye balance (CEB), and chair rises (CR) tests. For standing balance, we only used the closed-eye test because of the wider variability of the measurement results compared to the test with eyes open. For each of the three tests, the scoring ranged from 0 (lowest level) to 4 (highest level), based on performance percentile ranges. The GS test scoring was based on maximum grip strength achieved through the six measurements, with participants of each sex categorized into pentiles and the corresponding scores 0–4. As for the CEB test, we awarded a score of 4 to those who maintained the closed-eyes balance for 30 s, and the remaining participants received scores 0–3 according to quartiles of the balance time in the interval below the cut off. The CR test was scored from 4 to 1 based on the inverted to quartiles of the time taken to perform 10 chair rises, with a score of 0 assigned to those unable to perform 10 rises. The detailed description of the scoring for each test score is presented in Supplementary Table S2 and the scoring thresholds are shown in Table 3. The resulting CPPS score, a sum of GS, CEB, and CR scores, ranged from 0 (the poorest performance) to 12 (the highest possible performance).

### Exposure variable

The alcohol exposure was a categorical variable dividing participants into four groups based on their self-reported drinking behavior in the preceding 12 months. People who reported no alcohol consumption were categorized as having non-drinking status. Non-problem drinking status was defined as consuming alcohol but scoring < 8 on the AUDIT test and < 2 on the CAGE test. Hazardous drinking was defined as consuming alcohol and scoring 8–15 on the AUDIT test and/or 2–3 on the CAGE test. Harmful drinking was defined as consuming alcohol and meeting at least one of the following criteria: a score ≥ 16 on the AUDIT test^45–48^; a score of 4 on the CAGE test^49,50^; reported “zapoi” event in the past 12 months, or experienced hangovers or excessive drunkenness ≥ 2 times per week in the past 12 months.

The similar categorization method was used in previous studies, which found significant differences between the groups on annual volume of alcohol consumption, seeking medical care for alcohol-related problems, and levels of blood biomarkers of alcohol drinking^51^. The applied categorization approach rests on using several measurements of alcohol exposure and reduces recall bias compared to using a single measure, such as the frequency-quantity method^52^.

### Confounders

Several variables were considered as potential confounders in this study: age (years), city of residence (Arkhangelsk or Novosibirsk), education level (incomplete secondary, secondary, higher), smoking status (never, past, or current), body mass index (BMI, kg/m^2^), height (cm), physical activity level according to the EPIC questionnaire (inactive, moderately inactive, moderately active, or active), diet quality assessed using the Dietary Quality Score questionnaire (unhealthy, average, or healthy), and presence of chronic diseases (yes/no). Having a chronic disease was defined as a self-reported medical history of arterial hypertension, myocardial infarction, heart failure, atrial fibrillation, angina pectoris, stroke, diabetes, kidney disease, chronic bronchitis, cancer, asthma, rheumatoid arthritis, or osteoarthritis.

### Statistical analyses

This study utilized counts and percentages for categorical variables and means (M) with standard deviations (SD) or medians (Me) with quartiles (Q1, Q3) for continuous variables depending on data distribution. Accordingly, we employed Pearson’s chi-squared test for comparing groups on categorical variables and ANOVA or Kruskal–Wallis test for continuous variables.

Multiple linear and ordinal regressions with stepwise adjustments for potential confounders (Models 1–5) were used to evaluate the relationship between the level of alcohol consumption and physical performance. Model 1 included adjustments for age, height, and city of residence; Model 2 was additionally adjusted for education level; Model 3 included additional adjustments for smoking status; Model 4 was further adjusted for body mass index (BMI), diet quality, and physical activity level; and Model 5 included the concluding adjustments for the presence of chronic diseases. Non-problem drinking was used as the reference group in all models. The adjusted beta coefficients and proportional odds ratios (OR) with 95% confidence intervals (CI) were used to present the results of linear and ordinal regressions, respectively. The assumptions of the linear models were estimated through visual assessments of the residual distributions.

To validate the newly developed Composite Physical Performance Score (CPPS), Cronbach’s Alpha was used to assess its internal consistency. The Spearman correlation coefficients were calculated to measure the associations between the scores of the three physical tests composing the CPPS.

All statistical analyses were performed using Stata 17.0 software (StataCorp, College Station, Texas, USA).

### Ethical approval

The Know Your Heart study was approved by the ethics committees of the London School of Hygiene and Tropical Medicine (approval number 8808; received 24 February 2015), Novosibirsk State Medical University (approval number 75; received 21 May 2015), the Research Institute of Internal and Preventative Medicine, Novosibirsk (no approval number; received 26 December 2014), and the Northern State Medical University, Arkhangelsk (approval number 01/01-15; received 27 January 2015).

### Informed consent

All participants signed an informed consent form.

## Results

### Alcohol use characteristics

The study included 4215 participants, comprising 2200 Arkhangelsk residents (919 men and 1281 women) and 2015 Novosibirsk residents (840 men and 1175 women). Among men, drinking status was as follows 13.3% (234) non-drinking, 54.4% (954) non-problem drinking, 23.9% (421) hazardous drinking, and 8.5% (150) harmful drinking. Among women, the percentages were 10.5% (257), 83.8% (2059), 5.2% (127), and 0.5% (13), respectively.

The proportions of participants drinking alcohol at least twice per week among men increased from non-problem drinking (15.6%) to hazardous (38.7%) and harmful drinking (36.7%) (Table 1). Among women, the same pattern was observed, with the proportion rising from 2.6% with non-problem drinking to 13.4% and 7.7% with hazardous and harmful drinking, respectively.

**Table 1:** Alcohol-related characteristics by levels of alcohol consumption, by sex.

Characteristics	Men (N = 1759)				*p*^c^	Women (N = 2456)				*p*^c^
	Non-drinking	Non-problem drinking	Hazardous drinking	Harmful drinking		Non-drinking	Non-problem drinking	Hazardous drinking	Harmful drinking	
N (%)	234 (13.3)	954 (54.2)	421 (23.9)	150 (8.5)		257 (10.5)	2059 (83.8)	127 (5.2)	13 (0.5)	
	N (%)					N (%)				
Drinking alcohol ≥ 2 times/week	–	148 (15.6)	163 (38.7)	55 (36.7)	< 0.001	–	55 (2.6)	17 (13.4)	< 5^d^	< 0.001
Episodes of “zapoi”	–	–	–	106 (70.7)	–	–	–	–	9 (69.2)	–
Hangover ≥ 2 times/week	–	–	–	9 (6.0)	–	–	–	–	< 5^d^	–
Excessive drunkenness ≥ 2 times/week	–	–	–	6 (4.0)	–	–	–	–	< 5^d^	–
	Me (Q1; Q3)					Me (Q1; Q3)				
AUDIT, score	0 (0; 0)	3 (2; 5)	8 (7; 10)	13 (8; 16)	< 0.001	0 (0; 0)	2 (1; 3)	5 (3; 8)	12 (5;17)	< 0.001
CAGE, score	0 (0; 0)	0 (0; 0)	2 (1; 2)	3 (2; 4)	< 0.001	0 (0; 0)	0 (0; 0)	2 (2; 2)	3 (2; 3)	< 0.001
Annual pure alcohol consumption, L/y	0 (0; 0)	2.0 (0.9; 5.1)	7.0 (3.3; 14.5)	9.2 (3.4; 23.6)	< 0.001	0 (0; 0)	0.5 (0.2; 1.2)	2.9 (1.5; 6.7)	3.3 (2.0; 6.5)	< 0.001
CDT^a^, %	0.6 (0.5;0.7)	0.8 (0.6;1.0)	0.9 (0.7;1.3)	1.2 (0.7;2.4)	< 0.001	0.6 (0.4; 0.7)	0.7 (0.5; 0.8)	0.7 (0.5; 1.0)	0.8 (0.5; 0.9)	< 0.001
GGT^b^, U/L	25.0 (18.0; 39.8)	29.3 (21.2; 44.1)	41.4 (26.5; 66.7)	41.6 (28.5; 68.4)	< 0.001	20.7 (14.9; 29.9)	20.1 (15.0; 30.0)	24.3 (15.7; 40.6)	27.0 (20.7; 40.9)	0.008

^a^Carbohydrate-deficient transferrin, assessed in 1670 participants only (non-drinking: 166, non-problem drinking: 833, hazardous drinking: 537, harmful drinking: 144).

^b^Gamma-glutamyl transferase.

^c^Pearson’s chi-squared test for categorical variables, Kruskal–Wallis test for continuous variables.

^d^Numbers redacted due to cell counts less than 5 to preserve data anonymity.

The volume of alcohol consumption increased significantly in the same direction across the study groups. Among men, the median annual consumption ranged from 2.0 L for non-problem drinking group to 9.2 L in the harmful drinking group. Among women, the median values varied from 0.5 to 3.3 L in the corresponding groups.

The median values of CDT and GGT increased with increasing alcohol consumption levels from the non-problem drinking to the harmful drinking group in men (CDT: from 0.6 to 1.2%, GGT: from 25.0 to 41.6 U/L) and women (CDT: from 0.6 to 0.8%, GGT: from 20.1 to 27.0 U/L).

### Sociodemographic, behavioral and health-related characteristics

The median age of participants was 55 years for men and 54 years for women. The non-drinking group had higher median age compared to other groups for both sexes (58 years), while the hazardous and harmful drinking groups were younger (men: 51 and 53 years, women: 49 and 48 years) (Table 2).

**Table 2:** Sociodemographic, behavioral and health-related characteristics by levels of alcohol consumption, by sex.

Characteristics	Men (N = 1759)				*p*^a^	Women (N = 2456)				*p*^a^
	Non-drinking	Non-problem drinking	Hazardous drinking	Harmful drinking		Non-drinking	Non-problem drinking	Hazardous drinking	Harmful drinking	
N (%)	234 (13.3)	954 (54.2)	421 (23.9)	150 (8.5)		257 (10.5)	2059 (83.8)	127 (5.2)	13 (0.5)	
Age, years, Me (Q1; Q3)	58 (49; 63)	55 (47; 63)	51 (44; 60)	53 (45; 61)	< 0.001	58 (50; 65)	54 (45; 62)	49 (43; 54)	48 (43; 50)	< 0.001
Height, cm, M ± SD	173.9 ± 6.5	175.3 ± 6.8	175.6 ± 6.7	173.7 ± 6.9	< 0.001	160.3 ± 5.9	161.7 ± 6.2	162.3 ± 5.5	161.0 ± 6.9	0.004
BMI, kg/m^2^, M ± SD	27.3 ± 4.4	27.8 ± 4.7	28.1 ± 4.8	26.2 ± 4.8	< 0.001	28.7 ± 6.4	28.4 ± 5.9	28.0 ± 5.9	28.0 ± 8.6	0.768
	N (%)					N (%)				
City of residence					< 0.001					0.009
Arkhangelsk	105 (44.9)	465 (48.7)	260 (61.8)	89 (59.3)		114 (44.4)	1081 (52.5)	77 (60.6)	> 5^b^	
Novosibirsk	129 (55.1)	489 (51.3)	161 (38.2)	61 (40.7)		143 (55.6)	978 (47.5)	50 (39.4)	< 5^b^	
Education level					< 0.001					< 0.001
Primary	28 (12.0)	65 (6.8)	34 (8.1)	18 (12.0)		21 (8.2)	93 (4.5)	10 (7.9)	< 5^b^	
Secondary	142 (60.7)	433 (45.4)	216 (51.3)	95 (63.3)		147 (57.2)	1053 (51.1)	76 (59.8)	10 (76.9)	
Higher	64 (27.3)	456 (47.8)	171 (40.6)	37 (24.7)		89 (34.6)	913 (44.4)	41 (32.3)	< 5^b^	
Smoking status					< 0.001					< 0.001
Never	64 (27.4)	325 (34.1)	92 (21.9)	15 (10.0)		174 (67.7)	1465 (71.2)	40 (31.5)	< 5^b^	
In the past	87 (37.2)	351 (36.8)	148 (35.2)	45 (30.0)		40 (15.6)	303 (14.8)	28 (22.1)	< 5^b^	
Current smoker	83 (35.5)	278 (29.1)	181 (43.0)	90 (60.0)		43 (16.7)	290 (14.0)	59 (46.4)	11 (84.6)	
Diet quality					0.005					0.483
Unhealthy	37 (16.0)	103 (10.9)	47 (11.2)	24 (16.0)		24 (9.4)	146 (7.1)	13 (10.2)	< 5^b^	
Average	152 (65.8)	650 (68.6)	294 (70.0)	114 (76.0)		161 (62.9)	1300 (63.3)	83 (65.4)	8 (61.5)	
Healthy	42 (18.2)	195 (20.6)	79 (18.8)	12 (8.0)		71 (27.7)	608 (29.6)	31 (24.4)	< 5^b^	
Physical activity					0.382					0.001
Inactive	10 (4.3)	52 (5.5)	20 (4.8)	8 (5.4)		5 (2.0)	145 (7.2)	10 (8.0)	< 5^b^	
Moderately inactive	17 (7.3)	92 (9.8)	41 (9.9)	20 (13.5)		24 (9.6)	196 (9.7)	13 (10.4)	< 5^b^	
Moderately active	119 (51.3)	522 (55.6)	218 (52.7)	77 (52.0)		171 (68.7)	1213 (59.8)	63 (50.4)	< 5^b^	
Active	86 (37.1)	273 (29.1)	135 (32.6)	43 (29.1)		49 (19.7)	474 (23.3)	39 (31.2)	6 (46.1)	
Chronic diseases, yes	174 (74.4)	662 (69.4)	289 (68.7)	104 (69.3)	0.446	221 (86.0)	1566 (76.1)	98 (77.2)	12 (92.3)	0.002

^a^ANOVA or Kruskal–Wallis test for continuous variables, Pearson’s chi-squared test for categorical variables.

^b^Numbers redacted due to cell counts less than 5 to preserve data anonymity.

The hazardous drinking group had the highest mean heights for both men (175.6 cm) and women (162.3 cm) compared to other groups. Among men, the highest mean BMI was also in the hazardous drinking group (28.1 kg/m^2^) while it was the lowest in the harmful drinking group (26.2 kg/m^2^), and no significant differences were observed among women.

Higher proportions of hazardous and harmful drinking groups resided in Arkhangelsk, and less in Novosibirsk. Higher education was most common among the non-problem drinking group (men: 47.8%, women: 44.4%), while secondary education was most prevalent among the harmful drinking group (men: 63.3%, women: 76.9%). Current smoking was more prevalent among the harmful drinking group (men: 60.0%, women: 84.6%) compared to the non-problem drinking group (men: 29.1%, women: 14.1%).

Dietary quality differed between the compared group only among men, with the non-drinking and harmful drinking groups both having the highest proportions of those with unhealthy diets (16.0% each). Physical activity did not differ in men, but in women, those in the harmful drinking group were more likely to be physically active (46.1%) than women in the non-drinking group (19.7%). Lastly, chronic diseases were most commonly self-reported by women who were in the non-drinking (86.0%) and harmful drinking groups (92.3%).

### Construction and validation of the composite physical performance scale

The threshold values of the three functional tests constructing the CPPS and their corresponding scores are presented in Table 3. The scores of the tests correlated significantly. Specifically, the Spearman correlation coefficients were as follows: CR and CEB were correlated at 0.27; CR and GS were correlated at 0.20; and CEB and GS had a correlation of 0.27. All of these correlations were significant at *p* < 0.001. The CPPS ranged from 0 to 12 and had the mean value of 6.3, the median of 6, and the interquartile range of 4. The Cronbach’s alpha was 0.59.

**Table 3:** Thresholds of the functional tests constructing the Composite Physical Performance Scale (CPPS).

Score	Grip strength (kg)		Closed-eyes balance (sec)	Chair rises test (sec)
	Men	Women		
0	14.2–41.8	6.4–24.2	0–3.0	< 10 rises
1	41.9–46.6	24.3–27.4	3.1–5.4	27.7–81.0
2	46.7–51.1	27.5–30.0	5.5–11.1	24.2–27.6
3	51.2–56.0	30.1–33.5	11.2–29.5	21.1–24.1
4	56.1–88.6	33.6–51.2	≥ 30	8.0–21.0

### Association of alcohol consumption and score of the composite physical performance scale

In men, the CPPS mean scores, adjusted for age, height, and city of residence, were lower in the non-drinking (− 0.90, 95% CI − 1.24; − 0.57), hazardous drinking (− 0.27, 95% CI − 0.54; − 0.01), and harmful drinking groups (− 1.19, 95% CI − 1.59; − 0.70) than in the non-problem drinking group (Table 4). After adjustment for education, these differences attenuated but remained significant for non-drinking (− 0.71, 95% CI − 1.04; − 0.38) and harmful drinking (− 0.98, 95% CI: − 1.37; − 0.58). Further adjustments for smoking status, BMI, diet quality, physical activity, and chronic diseases had minor impact, sustaining the differences between non-drinking (− 0.65, 95% CI − 0.98; − 0.32) and harmful drinking groups (− 0.73, 95% CI − 1.13; − 0.33) compared to non-problem drinking.

**Table 4:** Linear regression on relationship of alcohol consumption and score of the Composite Physical Performance Scale (CPPS), by sex.

	Men (N = 1759)				Women (N = 2456)			
	Non-drinking	Non-problem drinking	Hazardous drinking	Harmful drinking	Non-drinking	Non-problem drinking	Hazardous drinking	Harmful drinking
	B (95%CI)				B (95%CI)			
Model 1^a^	**− 0.90 (− 1.24; − 0.57)**	Ref	**− 0.27 (− 0.54; − 0.01)**	**− 1.19 (− 1.59; − 0.79)**	**− 0.42 (− 0.70; − 0.13)**	Ref	0.16 (**− **0.23; 0.55)	**− 2.05 (− 3.24; − 0.87)**
Model 2^b^	**− 0.71 (− 1.04; − 0.38)**	Ref	**− **0.20 (**− **0.46; 0.06)	**− 0.98 (− 1.37; − 0.58)**	**− 0.37 (− 0.65; − 0.09)**	Ref	0.26 (**− **0.13; 0.65)	**− 1.79 (− 2.97; − 0.61)**
Model 3^c^	**− 0.70 (− 1.02; − 0.37)**	Ref	**− **0.13 (**− **0.39; 0.13)	**− 0.83 (− 1.23; − 0.43)**	**− 0.36 (− 0.64; − 0.08)**	Ref	0.37 (**− **0.02; 0.77)	**− 1.54 (− 2.3; − 0.36)**
Model 4^d^	**− 0.67 (− 1.00; − 0.34)**	Ref	**− **0.14 (**− **0.40; 0.13)	**− 0.75 (− 1.15; − 0.35)**	**− 0.38 (− 0.67; − 0.09)**	Ref	0.35 (**− **0.05; 0.75)	**− 1.59 (− 2.78; − 0.41)**
Model 5^e^	**− 0.65 (− 0.98; − 0.32)**	Ref	**− **0.12 (**− **0.39; 0.14)	**− 0.73 (− 1.13; − 0.33)**	**− 0.36 (− 0.65; − 0.08)**	Ref	0.37 (**− **0.03; 0.76)	**− 1.53 (− 2.71; − 0.34)**

Significant coefficients are marked in bold.

^a^Model 1 was adjusted for age, height, and city of residence.

^b^Model 2 was adjusted as per Model 1 plus education level.

^c^Model 3 was adjusted as per Model 2 plus smoking status.

^d^Model 4 was adjusted as per Model 3 plus BMI, diet quality, physical activity.

^e^Model 5 was adjusted as per Model 4 plus the presence of chronic diseases.

As for women, the adjusted CPPS mean scores were also lower in the non-drinking (− 0.42, 95% CI − 0.70; − 0.13) and harmful drinking groups (− 2.05, 95% CI − 3.24; − 0.87) compared to the non-problem drinking group. With stepwise adjustment for education, smoking status, BMI, diet quality, physical activity, and chronic diseases, the differences reduced insignificantly for both non-drinking (− 0.36, 95% CI − 0.65; − 0.08) and harmful drinking (− 1.53, 95% CI − 2.71; − 0.34) groups.

The difference in CPPS scores between women in the harmful drinking group and women in the non-problem drinking group (− 1.53) tended to be larger than the analogue difference in men (− 0.73), both being statistically significant regardless of the smaller size of the harmful-drinking group in women (N = 12) and the smaller power of the comparison.

### Association of alcohol consumption with grip strength, closed-eyes balance and chair rises scores

For GS, men in both the non-drinking and harmful drinking groups exhibited lower odds of having higher scores compared to men in the non-problem drinking group (Odds ratio (OR) = 0.52 (95% CI 0.40; 0.68) and OR = 0.53 (95% CI 0.38; 0.73), respectively), after adjusting for age, height, and city of residence (Table 5). These odds slightly decreased to 0.60 (95% CI 0.45; 0.79) for non-drinking and 0.67 (95% CI 0.48; 0.94) for harmful drinking after further adjustments. Similarly, women in the non-drinking and harmful drinking groups also had reduced odds of the higher GS scores after adjusting for age, height, and city of residence (OR = 0.76 (95% CI 0.60; 0.97) and OR = 0.26 (95% CI 0.10; 0.65), respectively). These odds remained relatively stable after the further adjustments (OR = 0.78 (95% CI 0.66; 0.99) for non-drinking and OR = 0.24 (95% CI 0.09; 0.60) for harmful drinking).

**Table 5:** Ordinal logistic regressions describing the associations of alcohol consumption with grip strength, closed-eyes balance, and chair rises scores, constructing of the Composite Physical Performance Scale (CPPS), by sex.

	Men (N = 1759)				Women (N = 2456)			
	Non-drinking	Non-problem drinking	Hazardous drinking	Harmful drinking	Non-drinking	Non-problem drinking	Hazardous drinking	Harmful drinking
	OR (95% CI)				OR (95% CI)			
Grip strength score (GS, 0–4)								
Model 1	**0.52 (0.40; 0.68)**	1.00	1.04 (0.84; 1.28)	**0.53 (0.38; 0.73)**	**0.76 (0.60; 0.97)**	1.00	1.25 (0.91; 1.73)	**0.26 (0.10; 0.65)**
Model 2	**0.56 (0.43; 0.74)**	1.00	1.06 (0.86; 1.31)	**0.57 (0.41; 0.79)**	**0.76 (0.60; 0.96)**	1.00	1.24 (0.90; 1.72)	**0.25 (0.10; 0.63)**
Model 3	**0.56 (0.43; 0.74)**	1.00	1.09 (0.88; 1.35)	**0.60 (0.43; 0.84)**	**0.76 (0.60; 0.96)**	1.00	1.21 (0.87; 1.69)	**0.24 (0.10; 0.61)**
Model 4	**0.59 (0.45; 0.78)**	1.00	1.01 (0.81; 1.25)	**0.66 (0.48; 0.92)**	**0.78 (0.61; 0.99)**	1.00	1.23 (0.88; 1.71)	**0.23 (0.09; 0.59)**
Model 5	**0.60 (0.45; 0.79)**	1.00	1.02 (0.82; 1.26)	**0.67 (0.48; 0.94)**	**0.78 (0.66; 0.99)**	1.00	1.23 (0.88; 1.72)	**0.24 (0.09; 0.60)**
Closed-eyes balance score (CEB, 0–4)								
Model 1	**0.74 (0.57; 0.96)**	1.00	**0.73 (0.60; 0.90)**	**0.55 (0.40; 0.75)**	0.85 (0.67; 1.08)	1.00	0.80 (0.58; 1.12)	**0.29 (0.11; 0.79)**
Model 2	0.83 (0.64; 1.07)	1.00	**0.76 (0.62; 0.94)**	**0.62 (0.45; 0.86)**	0.86 (0.68; 1.09)	1.00	0.86 (0.62; 1.20)	**0.34 (0.13; 0.92)**
Model 3	0.84 (0.65; 1.09)	1.00	0.81 (0.66; 1.00)	**0.70 (0.51; 0.97)**	0.87 (0.69; 1.11)	1.00	0.97 (0.69; 1.35)	0.42 (0.15; 1.15)
Model 4	0.84 (0.65; 1.10)	1.00	0.84 (0.68; 1.04)	**0.69 (0.49; 0.96)**	0.82 (0.64; 1.05)	1.00	0.98 (0.69; 1.38)	**0.36 (0.13; 0.98)**
Model 5	0.85 (0.66; 1.11)	1.00	0.85 (0.69; 1.05)	**0.70 (0.50; 0.97)**	0.83 (0.65; 1.06)	1.00	0.99 (0.70; 1.39)	0.37 (0.14; 1.01)
Chair rises score (CR, 0–4)								
Model 1	**0.60 (0.46; 0.78)**	1.00	0.89 (0.72; 1.10)	**0.56 (0.41; 0.77)**	0.83 (0.65; 1.06)	1.00	1.12 (0.81; 1.54)	0.70 (0.25; 1.98)
Model 2	**0.67 (0.51; 0.88)**	1.00	0.93 (0.75; 1.15)	**0.64 (0.46; 0.88)**	0.88 (0.69; 1.13)	1.00	1.24 (0.90; 1.72)	0.95 (0.33; 2.73)
Model 3	**0.67 (0.52; 0.88)**	1.00	0.95 (0.77; 1.18)	**0.67 (0.48; 0.92)**	0.89 (0.70; 1.14)	1.00	1.38 (0.99; 1.92)	1.21 (0.42; 3.47)
Model 4	**0.66 (0.50; 0.86)**	1.00	0.94 (0.75; 1.17)	**0.68 (0.49; 0.94)**	0.89 (0.69; 1.14)	1.00	1.37 (0.98; 1.91)	1.20 (0.42; 3.43)
Model 5	**0.66 (0.51; 0.87)**	1.00	0.94 (0.76; 1.17)	**0.68 (0.49; 0.94)**	0.90 (0.70; 1.15)	1.00	1.39 (0.99; 1.95)	1.25 (0.44; 3.57)

Significant ORs are marked in bold.

For CEB, men in the non-drinking, hazardous drinking, and harmful drinking groups showed reduced odds of achieving higher scores compared to non-problem drinking, controlling for age, height, and city of residence (OR = 0.74 (95% CI 0.57; 0.96), OR = 0.73 (95% CI 0.60; 0.90), and OR = 0.55 (95% CI 0.40; 0.75), respectively). Further adjustments yielded significantly reduced odds of higher CEB scores only for harmful drinking [OR = 0.70 (95% CI 0.50; 0.97)]. Among women, only the harmful drinking group showed reduced odds of higher CEB scores [OR = 0.29 (95% CI 0.11; 0.79)], which remained borderline significant after accounting for other factors.

Regarding CR, men in the non-drinking and harmful drinking groups had lower odds of obtaining higher scores compared to non-problem drinking after adjusting for age, height, and city of residence (OR = 0.60 (95% CI 0.46; 0.78) and OR = 0.56 (95% CI 0.41; 0.77), respectively). Further adjustments resulted in the OR of 0.66 (95% CI 0.51; 0.87) for non-drinking and 0.68 (95% CI 0.49; 0.94) for harmful drinking. Notably, no significant differences in the CR scale were observed among women divided by level of alcohol consumption.

## Discussion

This study is the first of its kind to investigate the relationship between alcohol consumption and physical performance using a combination of three physical tests (grip strength, closed-eye balance, and chair rises tests) taken together in the proposed Composite Physical Performance Scale. Besides, no previous research has examined this relationship in the general population of Russian adults.

Our findings revealed that both non-drinking and harmful drinking were associated with lower CPPS scores. Among men, harmful drinking was negatively linked to scores of all the three physical function tests composing the CPPS. Non-drinking showed a significant negative association with GS and CR scores, but not with CEB scores. Among women, those in the harmful drinking group had decreased CPPS scores, a negative association with GS score and a borderline significant negative association with CEB score. The non-drinking group among women also displayed a significant negative association with GS score, while there were no significant associations with the CEB and CR scores.

Our finding of reduced physical performance among those in the harmful drinking group aligns with previous research indicating that individuals with higher alcohol exposure experience decreased muscle strength. For instance, the 2-year prospective study by Cui et al. discovered that high alcohol consumption (> 34 g/day) was associated with a greater decline in grip strength relative to non-drinking Japanese men (− 2.83 vs. − 0.80 kg)^28^. Similarly, Buchman et al. reported a negative association of problematic drinking with muscle and grip strength among older German men with diabetes^22^. Wang et al. identified a J-shaped dose–response relationship between alcohol consumption and functional limitations in older European men, with muscle strength mediating these relationships^10^. In Russia, Dissing et al. demonstrated that adult men from the Izhevsk Family Study who were abstainers or engaged in hazardous drinking had reduced self-reported physical health compared to those who drank moderately^53^. Combined with these findings, our study supported the hypothesis that alcohol consumption reduces muscle strength^54,55^.

Several mechanisms have been proposed to explain how alcohol consumption affects muscle strength. These mechanisms include the related physical inactivity^56^, malnutrition^57^, dehydration^58^, hormonal imbalances, and disruption of protein homeostasis^59^. Besides, alcohol use can directly impede muscle growth and repair, leading to a reduction in the cross-sectional area of muscle with type II fibers^60,61^. It can also suppress global protein synthesis, even in response to anabolic stimuli such as muscle contraction, growth factors, and nutrients. The inhibition of mammalian target of rapamycin kinase (mTOR) activity and the enhancement of muscle protein synthesis under other catabolic conditions may contribute to this suppression^62^, ultimately resulting in decreased muscle mass and strength^63^. However, these mechanisms may not fully explain our findings of steeper decreases in the CPPS, GS and CEB in harmful-drinking in women compared analogue decreases men. Recent studies have suggested that alcohol may hinder skeletal muscle protein synthesis^64^ and trigger inflammation^65^. Women generally have higher rates of muscle protein synthesis compared to men^66,67^, and female hormones may act as a protective factor against inflammatory processes and thus weaken the impact of alcohol on muscle strength^68^. Regarding harmful-drinking in women, it is also interesting that we did not see a reduction in chair rises (CR) performance, which was present in men. However, all our findings regarding harmful-drinking in women should be interpreted with caution because of the small sample size in this study (N = 13) and the small power of the related analyses.

In contrast to our findings, several previous studies reported divergent results. For example, Hu et al. used Russian population data from the HAPIEE project and showed that odds ratios of having physical limitations were the same in the heaviest drinking group compared to regular moderate drinking, but increased for those who did not drink (1.61)^24^. Lee et al.^29^ found in a Korean population that binge and heavy drinking was associated with lower odds of having weak grip strength compared to non-drinking among men. The authors considered the possibility of reverse causation due to improved sociodemographic and health-related characteristics among people who drink alcohol but found that adjusting for these factors did not significantly affect the results. Similarly, Kawamoto et al.^69^ reported that grip strength increased with higher daily alcohol consumption in Japanese adults. Shan et al.^70^ observed a positive relationship between alcohol intake and handgrip strength in Malaysians, although the drinking group in that study were younger than the abstaining group. Additionally, Molina-Hidalgo et al.^71^ found that short-term daily alcohol consumption did not impact physical performance in healthy young adults.

The differences between the findings of our study and the earlier research may arise from different categorizations of the participants on the alcohol use level. Our approach utilized elevated thresholds of alcohol abuse and dependency tests (AUDIT and CAGE) as indicators of harmful drinking patterns, which have been earlier linked to increased mortality^52^. Therefore, harmful drinking in our study could represent a higher alcohol exposure compared to binge and heavy drinking categories in the other studies^6,29,71^.

In our study population, harmful drinking was associated with low education, smoking, unhealthy diet, and chronic diseases, whereas non-problem drinking was associated with healthier lifestyles and higher levels of education. For this reason, we examined the relationship between alcohol use and physical performance controlling for these characteristics as potential confounders. The adjustments resulted only in slight attenuations of the differences between non-problem and harmful drinking in physical performance, reflecting marginal confounding effects However, there could be other confounders, such as occupation, medication use, drinking and eating habits, and other cultural and socioeconomic factors that we did not measure and could not account for. Besides, despite the attempts to adjust for physical activity and diet, these factors are difficult to measure accurately from self-reported questionnaire data, so residual confounding from these factors cannot be excluded.

Numerous studies have shown that long-term alcohol use can have detrimental effects on body balance function, leading to ataxia^72^. A recent investigation conducted by Müller-Oehring et al.^73^ has provided evidence that disorganized neural communication among cerebellar-cortical regions may be responsible for this effect. In conjunction, Carvalho et al.^74^ have demonstrated that alcohol misuse among the elderly can lead to the deterioration of functional capacity for walking and various motor abilities. Our study builds upon these findings by demonstrating reduced balance test performance scores among those in the harmful drinking group in both men and women. The negative effects of long-term alcohol consumption on balance can be caused to a variety of factors, including disrupted cerebellar-cortical neural communication, cerebellar damage, and hindered sensory-motor integration^72–75^. These results are in line with our findings on reduced CEB test with harmful drinking in men.

Our study revealed a decline in physical performance among those with non-drinking status in both sexes, which is consistent with previous research^24,76^. One explanation for this is the “sick quitter” effect, meaning that people stop drinking alcohol for health reasons^25^. In our study, the non-drinking group was older and had a higher prevalence of chronic diseases, smoking, and unhealthy diets compared to participants with non-problem drinking. Even after accounting for these variables, residual confounding could have influenced the results. For these reasons, the reduced physical performance among non-drinkers might be more reflective of their health and lifestyle characteristics rather than an effect of not consuming alcohol.

We also observed no significant difference in physical performance between participants with hazardous drinking and non-problem drinking status. One plausible explanation is that hazardous drinkers could have a health reserve that allows them to maintain a relatively high level of drinking without immediate health consequences. They could be in a phase where sufficient resilience has not yet allowed the high level of alcohol consumption to manifest in reduced physical performance. However, these explanations must be considered hypothetical, given the cross-sectional nature of our study design. This emphasizes the need for longitudinal studies to better understand the long-term effects of hazardous drinking.

Several strengths enhanced the validity of this investigation. These include the use of standardized physical function measurements administered by trained professionals, the well-defined categorization of participants based on levels of alcohol consumption, and the inclusion of data from two cities in Russia. The utilization of the combination of self-reported drinking characteristics and validated tools for assessing alcohol-related problems (AUDIT and CAGE tests) helped to minimize biases resulting from subjective reporting of alcohol consumption. Moreover, the study examined physical performance among individuals with similar alcohol exposure levels as those seeking treatment for alcohol-related problems, allowing for the assessment of at-risk populations^52^.

Our study has several limitations that must be considered when interpreting the findings. Firstly, the cross-sectional design did not allow establishing a causal relationship between alcohol consumption and reduced physical performance. Secondly, the use of self-reported alcohol consumption data and self-report-based tests could have introduced social desirability bias or under-reporting, potentially affecting the accuracy of the division of participants into the alcohol consumption groups. However, this problem was unlikely significant as our categorization was substantiated by the corresponding differences in biomarkers of alcohol exposure (CDT and GGT). Thirdly, we had no available data to differentiate lifelong never-drinking from past drinking and to assess the reasons for quitting drinking alcohol, which may have influenced the findings regarding non-drinking. Fourthly, the small number of women categorized in the hazardous and harmful drinking groups may have limited the power to detect differences between these groups and the non-problem drinking group among women. Additionally, the exclusion of certain participants from statistical analyses and the incomplete physical function examinations may have introduced biases. Response rates of 68% in Arkhangelsk and 41% in Novosibirsk could have also contributed to sampling bias.

In this paper, we presented and used the CPPS—a new and potentially promising tool for measuring physical performance. However, we must outline that the primary aim of the study was not to validate the novel instrument for the assessment of physical performance, but to explore the association between different levels of alcohol intake and physical performance using the available data, specifically focusing on upper muscle strength, coordination, and lower muscle strength. Further research could examine the long-term validity of the Composite Physical Performance Scale (CPPS) and assess its utility and clinical significance in diverse populations, including individuals of different ages and ethnicities.

Lastly, at the routine calibration one of the dynamometers used in Novosibirsk was found to be broken, systematically measuring 2.5 kg higher during a part of the study period. Although the dynamometers were later calibrated in the UK, the exact timing of the device’s breakdown remains uncertain. Nonetheless, the issue could not significantly affect the conclusions as they did not rest on the grip strength values alone, but on the composite physical function score. The statistical analysis was also repeated using only the Arkhangelsk sample, yielding similar results and suggesting a negligible effect of the problem on the findings.

Our findings support the importance of reducing harmful alcohol consumption to maintain physical function, prevent negative outcomes such as frailty, disability, and mortality^14,16^, and promote overall physical health. Exercise training programs can be a part of the rehabilitation programs for harmful drinkers under treatment to restore their muscular strength and overall physical health after the extensive alcohol exposure^77^.

## Conclusion

Our study demonstrated that harmful drinking is associated with lower physical performance. Reduced physical performance in terms of grip strength, balance and chair rising capacities was observed among the harmful-drinking group for men and with respect to grip strength among the harmful-drinking group for women. Non-drinking was also negatively associated with physical performance scores in both sexes, with grip strength and chair rises abilities reduced in men and only grip strength in women. Further research is needed to understand the sex-specific effects of alcohol on physical performance.

### Supplementary Information

Supplementary Information.

## Data Availability

The datasets generated during and/or analyzed during the current study are available in the International Project on Cardiovascular Disease in Russia (IPCDR) repository, https://metadata.knowyourheart.science/.
