# Supplementary material for: The relationship between physical performance and alcohol consumption levels in Russian adults
Source: Sci Rep. 2024 Jan 16;14:1417. doi: 10.1038/s41598-024-51962-3 (PMC10792008; doi:10.1038/s41598-024-51962-3)
Supplement: Supplementary file 1 — Supplementary Information. [file 41598_2024_51962_MOESM1_ESM.docx]

**Supplementary Materials**

**Supplementary Table S1** Description of physical examination tests procedures

| Test | Description |
| --- | --- |
| Grip strength | Participants’ strength of grip was assessed using the Jamar+ Digital Dynamometer, following the Southampton protocol. Participants were seated with their arm rested on the armrest and their elbow bent at a 90-degree angle, with the hand positioned with the thumb facing upward. Three measurements were taken three times for each hand, alternating between the left and right hands, with the participants being verbally encouraged to exert maximum effort during each measurement. The maximum value achieved from the six measurements was used in the analysis. |
| Balance test | The procedure was performed by asking a participant to stand with eyes open on one leg as long as possible, taking the other leg off the floor by a few centimeters. The participant had to be barefoot, stood on a hard carpet, and was allowed to move arms, bend knees and stoop to keep the balance. A digital stopwatch was used to measure the test time. The measurement procedure started after the participant took a stable standing position on one leg (left or right depending on participant’s choice) with support at the elbow by a trained nurse. After the participant reported readiness to start, the support was removed and the stopwatch started. When the balance was lost (the second leg touched the floor) or if maintained for 30 seconds, the test was stopped and the time of the test duration was recorded to the nearest 0.01 seconds. The same test procedure was then repeated with the participant’s eyes closed. |
| Chair rises test | The test was performed barefoot, using an immobile chair 43 cm high, a hard carpet on the floor in front of it, and a digital stopwatch. A participant was asked to perform ten chair rises starting from a seated position with timing stopped when the participant sat down for the 10th time, and the time to the nearest 0.1 second was recorded. Participants were instructed to keep their arms across their chest during the test. If a participant could not complete ten chair rises or spent more than 2 minutes on the task, the number of rises was recorded. |

**Supplementary Table S2** Scoring of physical performance tests

| Test | Description |
| --- | --- |
| Grip strength | Participants were divided into five subgroups based on their maximum values (kg) obtained from six measurements on both arms (three on each), separately for men and women. The subgroups were divided at the 20th, 40th, 60th, and 80th percentile values of the maximum grip strength achieved. Participants with scores below the 20th percentile were assigned a GS score of 0, while those with scores between the 20th and 40th percentile received a score of 1, and so on, up to a maximum score of 4. The final GS score for each participant ranged from 0 (lowest level) to 4 (highest level). |
| Balance test | For the balance function, we employed the closed-eyes standing balance test (CEB). A participant who reached the threshold of keeping the balance for 30 seconds or more while standing on one leg with eyes closed was assigned a maximum value of 4 on the CEB scale. Other participants were divided into four quartile subgroups based on the time (in seconds) they were able to stand on one leg with eyes closed. Participants with scores up to the 25th percentile received a CEB score of 0, while those with results falling between the 25th and 50th percentile, 50th and 75th percentile, 75th and 100th percentile received CEB scores of 1, 2, and 3, respectively. The final CEB score for each participant ranged from 0 (lowest level) to 4 (highest level). |
| Chair rises test | A participant who could not complete 10 chair rises within two minutes received a score of 0. The remaining participants were divided into four quartile subgroups based on the time (in seconds) taken to complete 10 chair rises. Participants with scores up to the 25th percentile received a CR score of 4, while those in the 25th to 50th percentile, 50th to 75th percentile, and 75th to 100th percentile received scores of 3, 2, and 1, respectively. The final CR score for each participant ranged from 0 (lowest level) to 4 (highest level). |
